# Endoscopic Characteristics of Early Gastroesophageal Junction Adenocarcinomas and Assessment for Invasion Depth: A Case Series Study

**DOI:** 10.5152/tjg.2024.23312

**Published:** 2024-01-01

**Authors:** Shunzhe Song, Feng Yan, Jingwen Zhang, Aixia Gong

**Affiliations:** Department of Digestive Endoscopy, The First Affiliated Hospital of Dalian Medical University, Dalian, Liaoning Province, China

**Keywords:** Early adenocarcinoma, endoscopic characteristics, gastroesophageal junction, GEJ, invasion depth

## Abstract

**Background/Aims:**

Early-stage gastroesophageal junction (GEJ) adenocarcinoma can be challenging to diagnose and treat promptly using endoscopy. This study aims to summarize the endoscopic characteristics of early GEJ adenocarcinoma and investigate their correlation with pathological grade and invasion depth.

**Materials and Methods:**

This retrospective case series study evaluated patients with early GEJ adenocarcinoma who underwent endoscopic or surgical resection at First Affiliated Hospital of Dalian Medical University between January 2016 and December 2022.

**Results:**

A total of 71 patients were included in the analysis, with 59 males and a median age of 67 years. The majority of the lesions were located on the posterior side of the GEJ (40.8%) or the lesser curvature side (29.6%). Siewert II lesions accounted for 71.8% of cases, with most occurring on the posterior side (49.0%) and Siewert III lesions mostly occurring on the lesser curvature side (42.9%). Siewert I lesions accounted for only 7.0%, and all originated from Barrett mucosa. Paris classification of Is (*P* = .015) or IIc (*P* = .015), lesion size ≥12 mm (*P* = .017), red color with subsquamous extension (*P* = .038), and disordered microsurface with local fusion (*P* < .001) were independently and positively correlated with pathological grade and invasion depth by multivariable ordinal logistic regression.

**Conclusion:**

The posterior side and lesser curvature of the GEJ are the high-incidence sites of GEJ adenocarcinoma. Both forward and backward views during endoscopy should be combined to detect the lesion. Endoscopic characteristics such as Is or IIc morphology, larger size, red color with subsquamous extension, and disordered microsurface with local fusion may indicate a higher pathological grade and deeper invasion.

**Main Points**

- Early lesions of the gastroesophageal junction (GEJ) are concealed and difficult to detect. Improving the diagnostic rate of early GEJ adenocarcinoma is crucial for both survival rate and quality of life. However, there is a lack of reports regarding the endoscopic characteristics of early GEJ adenocarcinoma. The present study summarizes the endoscopic characteristics of GEJ adenocarcinoma.
- The study found that more than 70% of the lesions were located on the posterior side or lesser curvature of the GEJ. Nearly 70% of the lesions exhibited a reddish color, with or without subsquamous extension. Siewert II lesions were predominantly detected on the posterior side, while Siewert III lesions were mostly detected on the lesser curvature side. Siewert III lesions located at the lesser curvature always required detection through a backforward view using retroflexed endoscopy.
- Endoscopic ultrasound (EUS) is not accurate in assessing tumor depth at the GEJ, with a mere 48% concordance between EUS and pathological findings. Therefore, it is necessary to consider the endoscopic characteristics comprehensively. This study found that Is or IIc morphology, lesion size ≥12 mm, reddish color with subsquamous extension, and disordered microsurface with local fusion may indicate a deeper invasion depth.

## Introduction

Gastroesophageal junction (GEJ) adenocarcinoma accounts for approximately 30% of gastric cancer cases. A monitoring report of 42 tumors from 12 countries has shown a continuous increase in the incidence of GEJ adenocarcinoma since 2005, while the incidence of non-GEJ gastric cancer has gradually declined. Each year, around 260 000 patients are diagnosed with GEJ cancer.^1-3^ Patients diagnosed with advanced GEJ cancer often require proximal gastrectomy or even chemoradiotherapy. However, postoperative reflux symptoms can be persistent and significantly affect the quality of life.^4^ Early-stage GEJ adenocarcinoma can be completely removed using endoscopy while preserving the lower esophageal sphincter (LES). The success rate of a complete endoscopic resection is as high as 98.6%.^5^ Long-term follow-up studies have shown that endoscopic submucosal dissection (ESD) achieves similar survival rates compared to surgery.^6^

Due to its anatomical structure connecting the stomach and esophagus, the GEJ has unique characteristics. Early lesions of the GEJ are often concealed and difficult to detect, resulting in a missed diagnosis rate of more than 10%.^7^ Therefore, improving the diagnostic rate of early GEJ adenocarcinoma is crucial for both survival rate and quality of life. However, there is a lack of reports regarding the endoscopic characteristics of early GEJ adenocarcinoma. This study aims to summarize the endoscopic characteristics of GEJ adenocarcinoma and investigate their correlation with pathological grade and invasion depth.

## Materials and Methods

### Study Design and Case Selection

This retrospective case series study included 71 consecutive cases of early GEJ adenocarcinoma that underwent endoscopic or surgical resection at First Affiliated Hospital of Dalian Medical University between January 2016 and December 2022. GEJ was defined as cancer located within 5 cm proximal or distal to the Z line.^8^

Inclusion criteria: (1) Preservation of endoscopic images under a white light pattern, a narrow-band imaging (NBI) pattern, and a magnifying pattern was present. (2) Preoperative biopsy indicates that the lesion is dysplasia or carcinoma rather than inflammation. (3) Complete specimens were obtained, whether through endoscopic resection or surgical resection. Pathologists evaluated the cutting edge and basal edge of the specimens. (4) Pathological grade of resection specimen may be higher than that of preoperative biopsy. However, the carcinoma was limited to the mucosal or submucosal layer.

Exclusion criteria: (1) Patients underwent preoperative radiotherapy or chemotherapy. (2) The invasion depth of carcinoma exceeds the submucosal layer. (3) Pathological diagnosis indicates squamous carcinoma but not adenocarcinoma.

### Ethics Committee Approval

All procedures conducted in this study were in accordance with the 1964 Helsinki Declaration and approved by the Ethics Committee of First Affiliated Hospital of Dalian Medical University (grant number: PJ-KS-KY-2022-48). Informed consents were signed by the patients or their immediate family.

### Data Collection and Definition

Clinical data, including age, gender, Siewert classification, horizontal localization, endoscopic characteristics, and pathological assessment, were collected from medical records. The endoscopic characteristics of different Siewert subtypes were summarized, and the correlation between endoscopic characteristics and invasion depth was investigated.

Evaluation of endoscopic characteristics: Two experienced endoscopists evaluated all endoscopic images, including lesion size (>1.2 cm or ≤1.2 cm), Paris classification of lesion morphology (types I, IIa, IIb, and IIc), location of the GEJ (anterior side, posterior side, greater curvature side, and lesser curvature side), presence of Barrett mucosa, color features under white light pattern, and microsurface features under NBI and magnifying pattern. In case of disagreement between the 2 endoscopists, a third endoscopist made the final decision.

According to the “Siewert classification” standard,^9^ GEJ adenocarcinoma is divided into 3 types: Type I lesions are located 1-5 cm above the Z line; type II lesions are from 1 cm above to 2 cm below the Z line; and type III lesions are from 2 cm above to 5 cm below the Z line. Based on the horizontal localization of the lesion, it is divided into 4 directions: anterior side, posterior side, lesser curvature side, and greater curvature side.

Histopathological classification and invasion depth were diagnosed according to the Vienna classification of gastrointestinal epithelial neoplasia.^10^ Early GEJ adenocarcinoma was categorized into 4 types: (1) high-grade intraepithelial neoplasia (HGIN), (2) carcinoma in situ, (3) intramucosal carcinoma, and (4) submucosal invasive carcinoma. All pathological diagnoses were made by 2 independent pathologists, and in case of disagreement, a third pathologist intervened for the final diagnosis.

### Statistical Analysis

The data were analyzed using Statistical Package for the Social Sciences Statistics software, version 26.0 (IBM Corp.; Armonk, NY, USA). The correlation between count data was studied using the chi-square test. The correlation between ranked data was studied using the rank-sum test. Multivariable ordinal logistic regression was used to identify independent risk factors for statistically significant variables in univariate analysis. *P* <.05 was considered statistically significant.

## RESULTS

**Figure 1.**
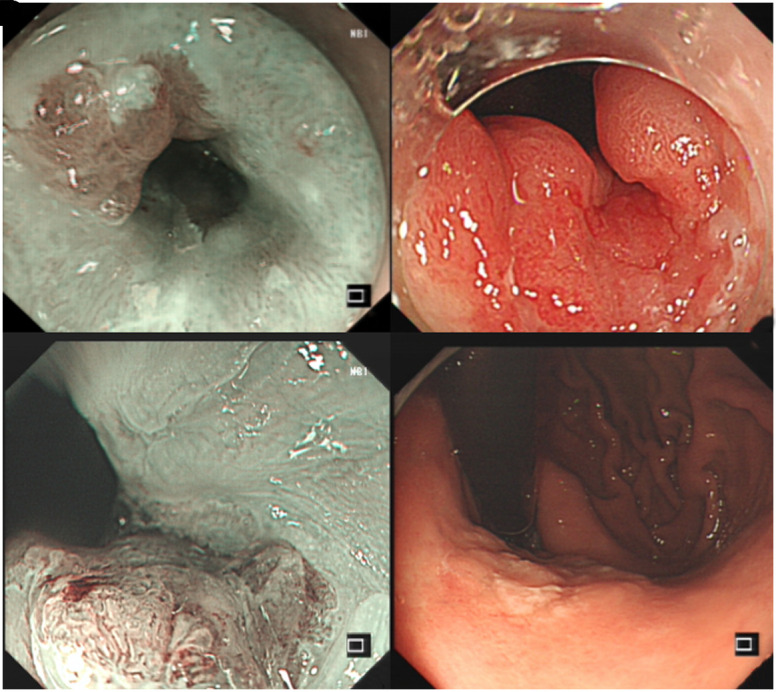
Endoscopic images of early GEJ adenocarcinoma of different Siewert classifications. (A) Siewert I: The lesion originated from Barrett esophageal mucosa. (B) Siewert II: The lesion exhibited a red and rough appearance (type IIb in the Paris classification) and was located on the posterior side of the GEJ. (C) Siewert II: The lesion exhibited a red and slightly protrusive appearance (type IIa) and was also located on the posterior side. (D) Siewert III: The lesion appeared slightly faded and flat (type IIb) and was located on the lesser curvature side.

A total of 71 consecutive cases were included in this study, comprising 59 males (83.1%) with a median age of 67 years (Table 1). Siewert II accounted for the highest proportion at 71.8%, followed by Siewert III at 21.1%. Siewert I accounted for the lowest proportion (7.0%), and all cases originated from Barrett mucosa. Most of the lesions were located on the posterior wall of the GEJ (40.8%) or the lesser curvature (29.6%). Siewert II lesions were predominantly located on the posterior side (49.0%), while Siewert III lesions were mostly located on the lesser curvature side (42.9%). Siewert I lesions exhibited a flat (40.0%) or protrusion (60.0%) appearance without any depression. Additionally, 69.0% of the lesions exhibited a reddish color, with or without subsquamous extension (Figure 1).

Univariate analysis showed that Paris classification (*P* < .001), lesion size (*P* = .045), color characteristics (*P* = .047), and microsurface in magnifying pattern (*P* < .001) were significantly correlated with pathological grade and invasion depth (Table 2). These factors were included in a multivariate ordinal logistic regression model to identify independent risk factors.

**Figure 2.**
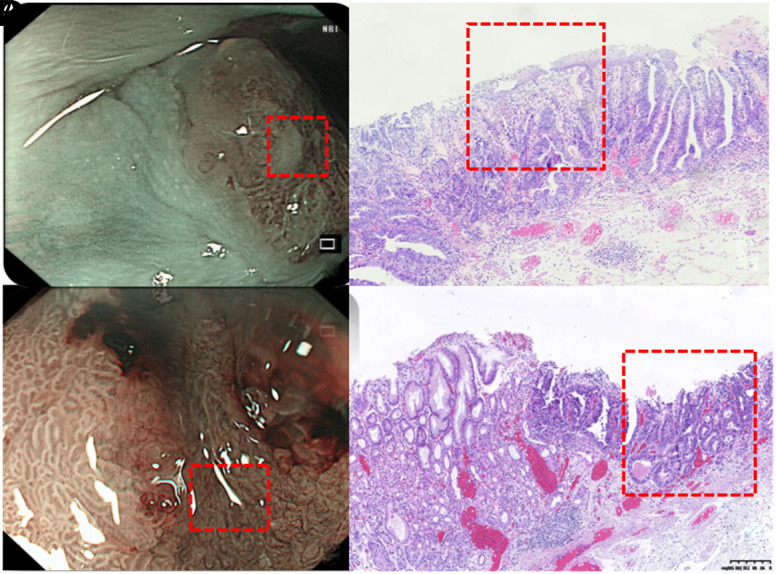
Endoscopic characteristics and pathological invasion depth. (A) The lesion exhibited a rough and red appearance with subsquamous extension. (B) Pathological section of (A): Cancerous glandular ducts were covered by a very thin squamous epithelium. (C) Under NBI and magnifying patterns the microsurfaces showed disorder, with shallow crypts suspected of local fusion. (D) Pathological section of (C): The microsurface suspected of fusion within the red frame of (C) was pathologically observed as densely arranged and disordered cancerous glandular ducts.

Multivariable ordinal logistic regression showed that the Paris classification of Is (*P* = .015) or IIc (*P* = .015), lesion size ≥12 mm (*P *= .017), red color with subsquamous extension (*P *= .038), and disordered microsurface with local fusion (*P* < .001) were independently and positively correlated with pathological grade and invasion depth (Table 3). As shown in Figure 2, a reddish color with subsquamous extension and a disordered microsurface of local fusion indicated deeper pathological invasion.

## Discussion

Early-stage GEJ adenocarcinoma lacks typical symptoms and is easily overlooked. Compared to intravenous anesthesia, conscious sedation allows patients to inhale deeply, providing better exposure to the GEJ mucosa.^11^ The present study summarized the endoscopic characteristics of early GEJ adenocarcinoma to improve the detection rate. In this study, 71.8% of the lesions were Siewert II. Similarly, Urabe et al^12^ found that 75 out of 103 GEJ adenocarcinoma were Siewert II. Besides, nearly 50% of Siewert II lesions were detected on the posterior side. Kariyawasam et al^13^ evaluated the circumferential distribution of Barrett’s neoplasia in 80 patients. The study showed that 53.8% of Barrett’s cancers and HGIN lesions were centered within an arc from 2 to 5 o’clock. Investigate the reason why the squamous epithelium in the posterior side is the most susceptible site to gastric acid damage.^14^ The present study found that most Siewert III lesions were detected on the lesser curvature side. Urabe et al^12^ also found that the background mucosa of type III lesions showed marked mucosal atrophy and intestinal metaplasia. According to Kimura–Takemoto classification, the atrophic boundary extends upward along the lesser curvature.^15^ This may explain why Siewert III lesions were predominantly detected on the lesser curvature side of GEJ. It is worth mentioning that lesions located at the lesser curvature always required detection through backforward view using retroflexed endoscopy.

In contrast to gastric cancer, GEJ adenocarcinoma, especially Barrett adenocarcinoma, carries a higher risk of lymph node metastasis. Leers et al^16^ found that the rate of lymphatic metastasis varies with the invasion depth, ranging from 1.3% in the mucosal layer to as high as 22% in the submucosal depth. Chevallay et al^17^ recommended endoscopic resection as an absolute indication for PT1aN0 GEJ adenocarcinoma. However, Kim et al^18^ found that for SM1 (submucosal infiltration depth <500 μm), there was no difference in survival rate between endoscopic resection and surgical resection as long as complete endoscopic resection was achieved. Of the 71 patients included in the present study, 8 patients lost contact during follow-up. Of the 63 patients with contact, 5 patients had local recurrence or lymph node metastasis during the follow-up, all of which were submucosal invasions. Considering the higher risk of lymph node metastasis, assessing the invasion depth is crucial for determining the resection method. Endoscopic resection only removes the lesion and the surrounding mucosa, preserving the integrity of the LES and the anti-reflux barrier. With the exception of individual cases of postoperative scar stricture,^19^ few serious complications have been reported. A systematic analysis^20^ demonstrated that the complete resection rate and en bloc resection rate through endoscopy were as high as 87.0% and 98.6%, respectively. Only 6.7% of patients experienced postoperative scar stenosis, which was relieved in all cases after balloon expansion.

Assessing the pathology and depth is equally important as detecting it, as determining the invasion depth is a key component in guiding the treatment strategy. Endoscopic ultrasound (EUS) is inaccurate in assessing tumor depth at the GEJ, with a mere 48% concordance between EUS and pathological findings.^21^ Therefore, it is necessary to consider the endoscopic characteristics comprehensively. This study found that Is or IIc morphology, lesion size ≥12 mm, reddish color with subsquamous extension, and disordered microsurface with local fusion may indicate a deeper invasion depth. Similar to our results, Takada et al^22^ found that noticeable depression or protrusion, lesion size ≥15 mm, and subepithelial extension of squamous epithelium were significantly correlated with lesion depth. Besides, a recent study^23^ showed that 44% of the patients had subsquamous extension. It is a critical characteristic of GEJ adenocarcinoma, which determines the lateral margin of the oral side.

In summary, there are 3 suggestions for endoscopists in the GEJ examination. First, pay attention to red coloration and rough surface, particularly on the posterior side and lesser curvature side. Secondly, combine forward and backward views to detect the Siewert III lesions located at the lesser curvature side. Lastly, consider that Is or IIc morphology, large size, reddish color with subsquamous extension, and disordered microsurface with local fusion may indicate a deeper invasion depth. This study has a notable limitation, as it is a single-center study with a small sample size of only 71 cases.

## Figures and Tables

**Table 1.** Clinical and Endoscopic Features of Early Gastroesophageal Junction Adenocarcinoma with Different Siewert Classification

	Type I (n = 5)	Type II (n = 51)	Type III (n = 15)
Gender			
Female	1	10	1
Male	4	41	14
Age (years)			
≤60	1	14	2
>60	4	37	13
Resection method			
Endoscopic resection	4	45	13
Surgical resection	1	6	2
Lesion size			
<1.2 cm	3	25	6
≥1.2 cm	2	26	9
Circumferential localization			
Anterior side	1	3	0
Posterior side	1	25	3
Greater curvature side	1	13	3
Lesser curvature side	2	10	9
Presence of Barrett mucosa			
Yes	5	8	0
No	0	43	15
Color characteristics			
Slightly faded or no obvious change	0	14	8
Reddish without subsquamous extension	5	27	7
Reddish with subsquamous extension	0	10	0
Paris classification			
Is	1	11	1
IIa	2	13	3
IIb	2	11	2
IIc	0	13	7
IIa + IIc	0	3	2

**Table 2.** Univariate Analysis of the Risk Factors for Predicting Pathological Grade and Invasion Depth

Pathological Type and Invasion Depth	HGIN	Carcinoma In Situ	Intramucosal Carcinoma	Submucosal Invasion	*χ* ^2^	*P*
Paris classification						
Is	1	1	5	6	35.690	.000
IIa	7	7	2	2		
IIb	9	6	0	0		
IIc	3	5	5	7		
IIa + IIc	0	1	0	4		
Siewert classification						
Type I	1	2	1	1	0.792	.997
Type II	14	14	9	14		
Type III	5	4	2	4		
Lesion size						
<1.2 cm	13	12	4	5	8.084	.044
≥1.2 cm	7	8	8	14		
Color characteristics						
Slightly faded or no obvious change	11	5	3	3	12.607	.047
Reddish without subsquamous extension	8	14	6	11		
Reddish with subsquamous extension	1	1	3	5		
Microsurface in magnifying pattern						
Disorder without fusion	20	13	7	6	20.275	.000
Disorder with suspected fusion	0	7	5	13		

HGIN, high-grade intraepithelial neoplasia.

**Table 3.** Ordinal Logistic Regression of the Risk Factors for Predicting Pathological Grade and Invasion Depth

Variables	Cases (n)	OR (95% CI)	*P*
Paris classification			
Iib	15	1 (reference)	
Iia	20	2.197 (0.552, 8.745)	.264
Iic	22	5.536 (1.395, 21.973)	.015
IIa + Iic	2	2.991 (0.153, 58.477)	.470
Is	12	7.801 (1.489, 40.859)	.015
Lesion size			
<1.2 cm	34	1 (reference)	
≥1.2 cm	37	3.197 (1.234, 8.282)	.017
Color characteristics			
Slightly faded or no obvious change	22	1(Reference)	
Reddish without subsquamous extension	39	1.810 (0.613, 5.342)	.282
Reddish with subsquamous extension	10	5.538 (1.098, 27.937)	.038
Microsurface in magnifying pattern			
Disorder without fusion	46	1 (reference)	
Disorder with suspected fusion	25	8.819 (2.884, 26.971)	.000
